# *CDCA8* as an independent predictor for a poor prognosis in liver cancer

**DOI:** 10.1186/s12935-021-01850-x

**Published:** 2021-03-08

**Authors:** Yu Shuai, Erxi Fan, Qiuyue Zhong, Qiying Chen, Guangyong Feng, Xiaoxia Gou, Guihai Zhang

**Affiliations:** 1 Department of Respiratory and Critical Care Medicine, Guizhou Aerospace Hospital, Zunyi, 563000 Guizhou People’s Republic of China; 2 Affiliated Hospital of Zunyi Medical University, Zunyi, 563000 Guizhou People’s Republic of China; 3 Department of Oncology, Zhuhai People’s Hospital (Zhuhai Hospital Affiliated with Jinan University), Zhuhai, 519000 Guangdong People’s Republic of China

**Keywords:** *CDCA8*, Liver cancer, Prognosis, The Cancer Genome Atlas

## Abstract

**Background:**

Human cell division cycle associated 8 (*CDCA8*) a key regulator of mitosis, has been described as a potential prognostic biomarker for a variety of cancers, such as breast, colon and lung cancers. We aimed to evaluate the potential role of *CDCA8* expression in the prognosis of liver cancer by analysing data from The Cancer Genome Atlas (TCGA).

**Methods:**

The Wilcoxon rank-sum test was used to compare the difference in *CDCA8* expression between liver cancer tissues and matched normal tissues. Then, we applied logistic regression and the Wilcoxon rank-sum test to identify the association between *CDCA8* expression and clinicopathologic characteristics. Cox regression and the Kaplan–Meier method were used to examine the clinicopathologic features correlated with overall survival (OS) in patients from the TCGA. Gene set enrichment analysis (GSEA) was performed to explore possible mechanisms of *CDCA8* according to the TCGA dataset.

**Results:**

*CDCA8* expression was higher in liver cancer tissues than in matched normal tissues. Logistic regression and the Wilcoxon rank-sum test revealed that the increased level of *CDCA8* expression in liver cancer tissues was notably related to T stage (OR = 1.64 for T1/2 vs. T3/4), clinical stage (OR = 1.66 for I/II vs. III/IV), histologic grade (OR = 6.71 for G1 vs. G4) and histological type (OR = 0.24 for cholangiocarcinoma [CHOL] vs. hepatocellular carcinoma [LIHC]) (all *P*-values < 0.05). Kaplan–Meier survival analysis indicated that high *CDCA8* expression was related to a poor prognosis in liver cancer (*P* = 2.456 × 10^−6^). Univariate analysis showed that high *CDCA8* expression was associated with poor OS in liver cancer patients, with a hazard ratio (HR) of 1.85 (95% confidence interval [CI]: 1.47–2.32; *P* = 1.16 × 10^–7^). Multivariate analysis showed that *CDCA8* expression was independently correlated with OS (HR = 1.74; CI: 1.25–12.64; *P* = 1.27 × 10^–5^). GSEA revealed that the apoptosis, cell cycle, ErbB, MAPK, mTOR, Notch, p53 and TGF-β signaling pathways were differentially enriched in the *CDCA8* high expression phenotype.

**Conclusions:**

High *CDCA8* expression is a potential molecular predictor of a poor prognosis in liver cancer.

## Background

Primary liver cancer is the sixth most common malignant tumour and the mortality caused by liver cancer ranks fourth in the world [1, 2]. There are approximately 841,000 new cases and 782,000 deaths worldwide each year, with a survival duration of only 6–20 months without any intervention [3]. Currently, surgical resection is still the main treatment method for liver carcinoma. Although increasing progress has been made in the diagnosis and treatment of liver cancer, owing to the metastasis and recurrence of liver cancer, the 5-year survival rate of patients is less than 8% [4]. Thus, more effective or novel tumour biomarkers that can be used to accurately diagnose and better predict prognosis in liver cancer are urgently needed.

The *CDCA8* gene encodes the Borealin/Dasra B protein and is a component of the chromosome passenger complex (CPC). The CPC is an important dynamic structure during cell division and consists of four parts: INCENP, Survivin, Aurora B and Borealin/Dasra B [5]. *CDCA8* plays critical roles in locating the CPC to the centromere, correcting kinetochore binding errors, and stabilizing bipolar spindles [6, 7]. Previous studies have reported *CDCA8* overexpression contributes to the proliferation of tumour cells, such as colorectal cancer and lung cancer cells [8, 9]. In addition, high *CDCA8* expression was found to represent a poor prognosis for gastric cancer [10]. However, the relationship between *CDCA8* expression and clinicopathological parameters in liver cancer is unclear.

In this study, we sought to use existing data from the TCGA to assess the value of *CDCA8* expression in liver cancer prognosis. Then, GSEA was performed to elucidate the biological pathways regulated by *CDCA8* that are involved in the pathogenesis of liver cancer. Ultimately, our results showed that increased *CDCA8* expression correlated with a poor prognosis in liver cancer. GSEA also indicated that the *CDCA8* high expression phenotype was related to the apoptosis, cell cycle, ErbB, MAPK, mTOR, Notch, p53 and TGF-β signaling pathways. We may find a novel biomarker of prognosis and potential molecular mechanisms that affect prognosis in liver cancer.

## Methods

### Data mining the TCGA database

*CDCA8* expression data (418 samples, Workflow Type: HTSeq-Counts) and corresponding clinical characteristic data were extracted from the official website of the TCGA liver cancer cohort (https://cancergenome.nih.gov/). In this study, we obtained the genomic expression information of *CDCA8* that was calculated by high-throughput sequencing from the TCGA database. Ethical approval was not required, as all are publicly available. After excluding normal liver tissues (58 samples), the expression differences according to discrete variables were visualized using boxplots [11]. Eventually, R software (version 3.5.1) was used to further analyse the RNA-Seq gene expression HTSeq-Counts data of liver cancer patients and clinical data.

### Gene set enrichment analysis (GSEA)

In the present research, the gene set “c2.cp.kegg.v6.2.symbols.gmt”, which served as a reference gene set, was downloaded from the Molecular Signatures Database (MSigDB) (http://software.broadinstitute.org/gsea/msigdb). We performed GSEA to reveal significant survival differences between the high and low *CDCA8* expression groups. Gene set arrangements were repeated 1,000 times for each analysis, and the expression level of *CDCA8* was treated as a phenotype label. We used the nominal P-value and normalized enrichment score (NES) to analyse pathway enrichment. The NES, enrichment score (ES), false discovery rate (FDR) and P-value were considered four key statistics in the GSEA. A gene set was considered significantly enriched when the P-value was less than 0.05 and the FDR was less than 0.25.

### Statistical analysis

Statistical analysis was performed using R (v.3.5.1). The Wilcoxon rank-sum test was used to compare the expression of *CDCA8* between the liver cancer and normal groups. We performed the Wilcoxon signed-rank test and logistic regression to estimate the relationship between *CDCA8* and clinicopathological variables. Subjects were divided into two groups according to the median value of gene expression, and patients with incomplete clinical data were excluded. We used Kaplan–Meier analysis to compare OS between the high and low *CDCA8* expression groups. Cox regression and the Kaplan–Meier method were used to examine the clinicopathological features correlated with OS in patients from the TCGA. P < 0.05 was considered statistically significant.

## Results

### Clinical characteristics of liver cancer patients in the TCGA

The characteristics of 418 patients with liver cancer, including sex, TNM classification, clinical stage, histological type, histologic grade, race, and vital status, were downloaded from the TCGA database (Table 1). In our study cohort, the median age at diagnosis was 61 years and ranged from 16 to 90 years. The median follow-up for subjects alive at last contact was 419 days and ranged from 0 to 3258 days.

**Table 1:** TCGA liver cancer patient characteristics

Characteristics	Number of sample, n (%)	
	n	Percentage (%)
Age (years)		
≤ 61	195	46.65
> 61	181	43.30
Unknown	42	10.05
Gender		
Male	272	65.07
Female	146	34.93
T stage		
T1	204	48.80
T2	107	25.60
T3	90	21.53
T4	14	3.35
TX	1	0.24
Unknown	2	0.48
N stage		
N0	290	69.38
N1	8	1.91
NX	119	28.47
M stage		
MO	303	72.49
M1	8	1.91
MX	107	25.60
Stage		
I	194	46.41
II	98	23.44
III	90	21.53
IV	12	2.87
Unknown	24	5.74
Histologic grade		
G1	55	13.16
G2	180	43.06
G3	124	29.67
G4	13	3.11
Unknown	46	11.00
Histological type		
LIHC	377	90.41
CHOL	41	9.59
Vital status		
Living	271	64.83
Deceased	147	35.17
Race		
White	222	53.11%
Not white	186	44.50%
Unknown	10	2.39%
Total	418	100.00%

*LIHC* Liver hepatocellular carcinoma, *CHOL* Cholangiocarcinoma, *TCGA* The Cancer Genome Atlas

## CDCA8 is highly expressed in liver cancer tissues

**Fig. 1:**
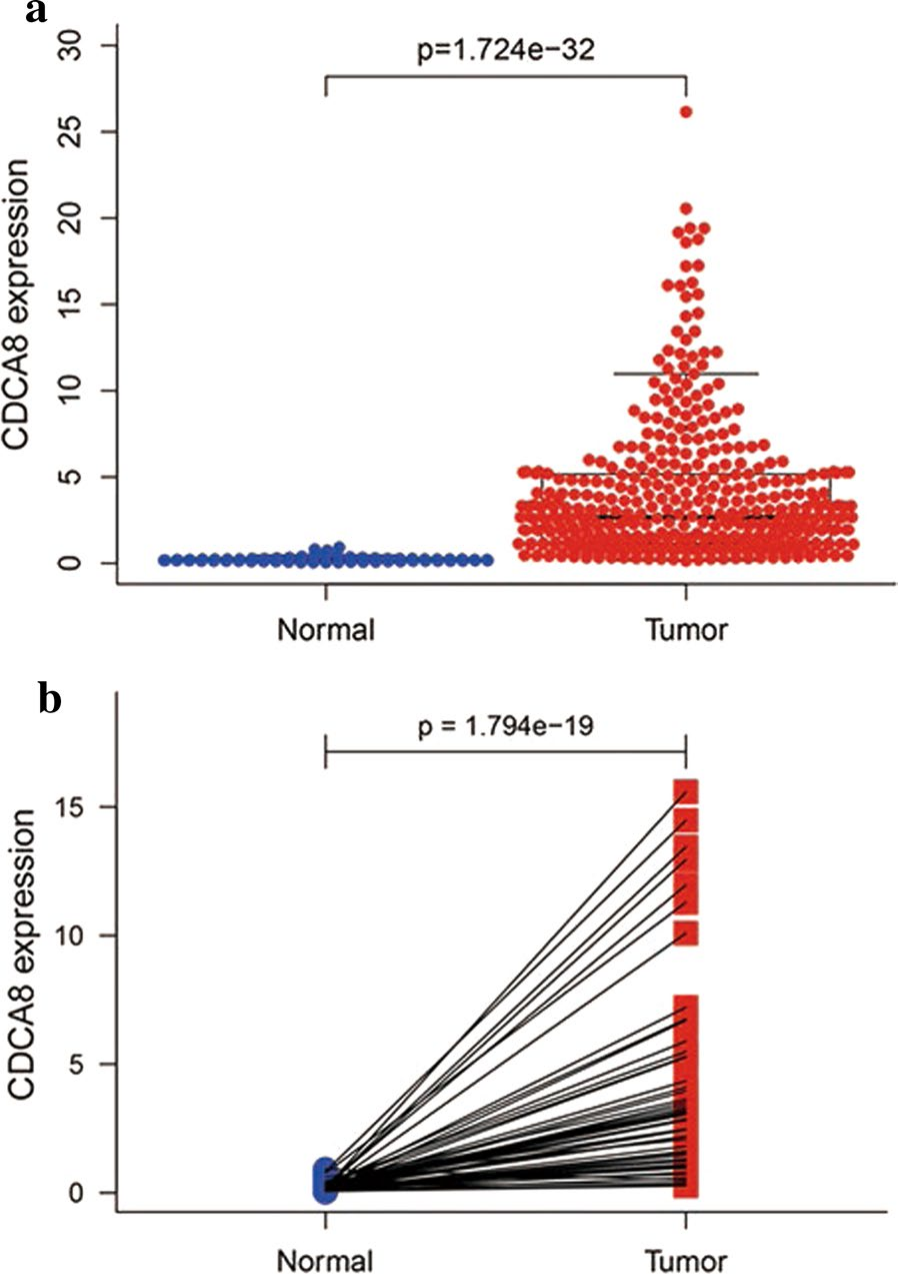
*CDCA8* expression is significantly higher in liver cancer tissues than in adjacent normal tissues. **a** *CDCA8* expression was significantly higher in cancer tissues than in normal tissues (P = 1.724e^−32^). **b** *CDCA8* expression was significantly higher in liver cancer tissues (P = 1.794e^−19^) than in 50 paired noncancerous adjacent tissues (Wilcoxon signed-rank test)

We used the Wilcoxon-rank sum test to analyse the relationship between *CDCA8* expression and different tissue characteristics, and the results showed that *CDCA8* expression was significantly higher in liver cancer tissues than in normal tissues (*P* = 1.724 × 10^−32^) (Fig. 1a). Subsequently, we used the Wilcoxon signed-rank test to determine *CDCA8* expression in 57 liver cancer tissues and matched adjacent normal tissues. *CDCA8* expression was significantly lower in normal tissues than in cancer tissues (*P* = 1.794 × 10^−19^)(Fig. 1b).

### Relationships between *CDCA8* expression and clinicopathological variables in liver cancer patients

**Fig. 2:**
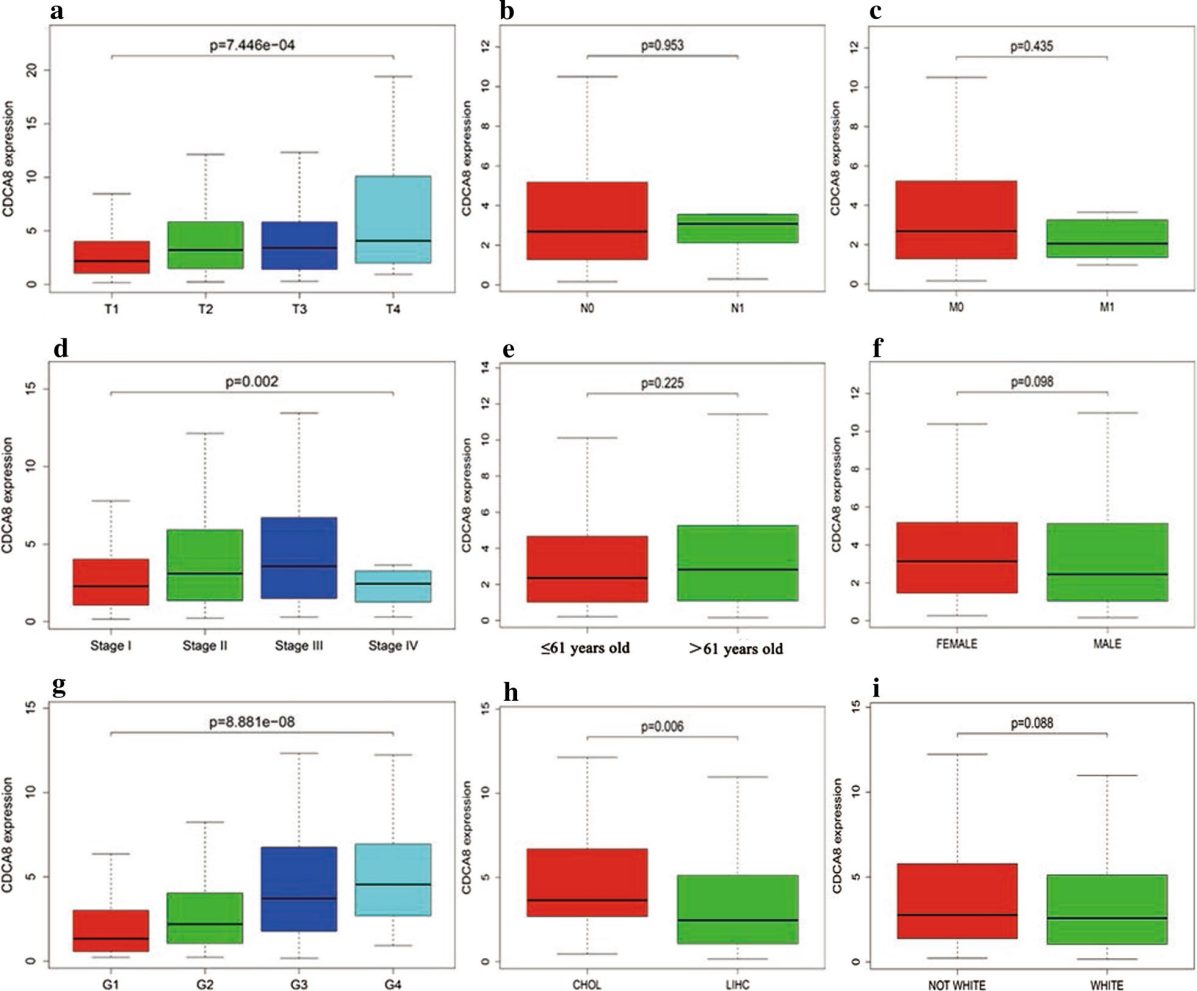
Relationship between *CDCA8* expression and clinicopathological characteristics. **a**–**c** TNM classification, **d** clinical stage, **e** age, **f** sex, **g** histologic grade, **h** histological type, and **i** race. LIHC, liver hepatocellular carcinoma; CHOL, cholangiocarcinoma; TCGA, The Cancer Genome Atlas; T, topography distribution; N, lymph node metastasis; M, distant metastasis

Logistic regression and the Wilcoxon rank-sum test revealed that the upregulation of *CDCA8* was obviously correlated with T stage (*P* = 7.446 × 10^−4^), clinical stage (*P* = 0.002), histologic grade (*P* = 8.881 × 10^−8^) and histological type (*P* = 0.006), as shown in Fig. 2. Afterward, univariate analysis using logistic regression was adopted to analyse the relationship between *CDCA8* expression (based on a median expression value of 2.64) and poor clinicopathologic variables. These results showed that high *CDCA8* expression was notably correlated with T stage (OR = 1.64 for T1/2 vs. T3/4), clinical stage (OR = 1.66 for I/II vs. III/IV), a high histologic grade (OR = 6.71 for G1 vs. G4), and histological type (OR = 0.24 for CHOL vs. LIHC) (Table 2), indicating that compared with patients with low *CDCA8* expression, those with high *CDCA8* expression tend to have a more advanced stage.

**Table 2:** *CDCA8* expression correlated with clinical pathological characteristics (logistic regression)

Clinical characteristics	Total (*N*)	Odds ratio in CDCA8 expression	*P-*value
T stage (T1/2 vs. T3/4 )	415	1.64 (1.03–2.62)	*0.035*
N stage (N0 vs. N1 )	298	3.11 (0.70-21.48)	0.169
M stage (M0 vs. M1 )	311	0.74 (0.14–3.44)	0.703
Clinical stage (I/II vs. III/IV)	394	1.66 (1.04–2.67)	*0.034*
Age (≤ 61 vs.> 61)	376	1.30 (0.86–1.95)	0.212
Gender (Female vs. Male)	418	0.70 (0.47–1.06)	0.095
Histologic grade (G1 vs.G4)	68	6.71 (1.76–33.18)	*0.009*
Histological type (CHOL vs. LIHC)	418	0.24 (0.10–0.54)	*0.001*
Race (Not white vs. White)	408	0.88 (0.594–1.31)	0.544

Categorical dependent variable, greater or less than the median expression level. Italic represents *P*‑values < 0.005. LIHC, Liver hepatocellular carcinoma; CHOL, Cholangiocarcinoma; T, topography distribution; N, lymph node metastasis; M, distant metastasis

### *CDCA8* may be an independent predictor of prognosis in liver cancer

**Fig. 3:**
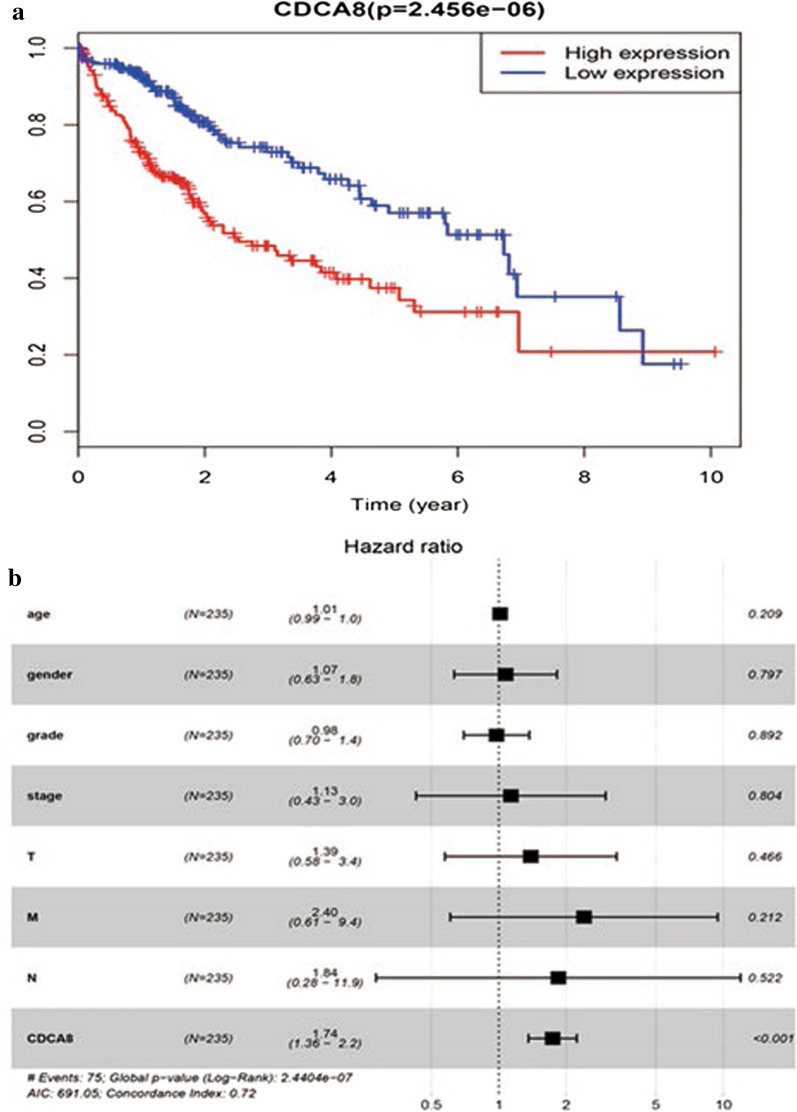
Survival outcomes and multivariate analysis. **a** The effect of *CDCA8* expression on overall survival in liver cancer patients in the TCGA cohort. The median score was used to divide patients into high expression and low expression groups. **b** A forest map of the results of the multivariate analysis. T, topography distribution; N, lymph node metastasis; M, distant metastasis

Kaplan–Meier survival analysis was performed to examine the role of *CDCA8* expression in predicting the prognosis of liver cancer patients. The results showed that patients with high *CDCA8* expression experienced a shorter OS duration than those with low *CDCA8* expression (*P* = 2.456 × 10^−6^)(Fig. 3a). Accordingly, we assessed the prognostic variables correlated with OS using univariate and multivariate Cox regression analyses (Table 3). The univariate Cox model revealed that high *CDCA8* expression was strongly associated with worse OS (HR = 1.85; 95% CI: 1.47–2.32; *P* = 1.16 × 10^–7^), as was clinical stage and T classification. As shown in Fig. 3b, high *CDCA8* expression was the only independent prognostic factor associated with OS (HR = 1.74; 95% CI: 1.36–2.23; *P* = 1.27 × 10^–5^) in the multivariate analysis.

**Table 3:** Univariate analysis and multivariate analysis of liver cancer patients overall survival

Characteristics	Univariate analysis			Multivariate analysis		
	HR	95 % CI	*P*	HR	95 % CI	*P*
T stage	1.8	1.43–2.27	*4.73 × 1*^*− 7*^	1.39	0.58–3.35	0.466
N stage	2.02	0.49–8.28	0.328			
M stage	3.85	1.21–12.28	0.022			
Clinical stage	1.86	1.46–2.39	*8.06 × 10*^*−7*^	1.13	0.43–2.99	0.804
Age	1.00	0.99–1.02	0.591			
Gender	0.78	0.49–1.25	0.301			
Histologic grade	1.02	0.75–1.39	0.914			
CDCA8	1.85	1.47–2.32	*1.16 × 10*− ^*7*^	1.74	1.36–2.23	*1.27 × 10*−^*5*^

Italic values indicate *P*‑values < 0.05, HR, hazard ratio; CI, confidence interval; T, topography distribution; N, lymph node metastasis; M, distant metastasis

### *CDCA8*-related signaling pathways according to GSEA

**Fig. 4:**
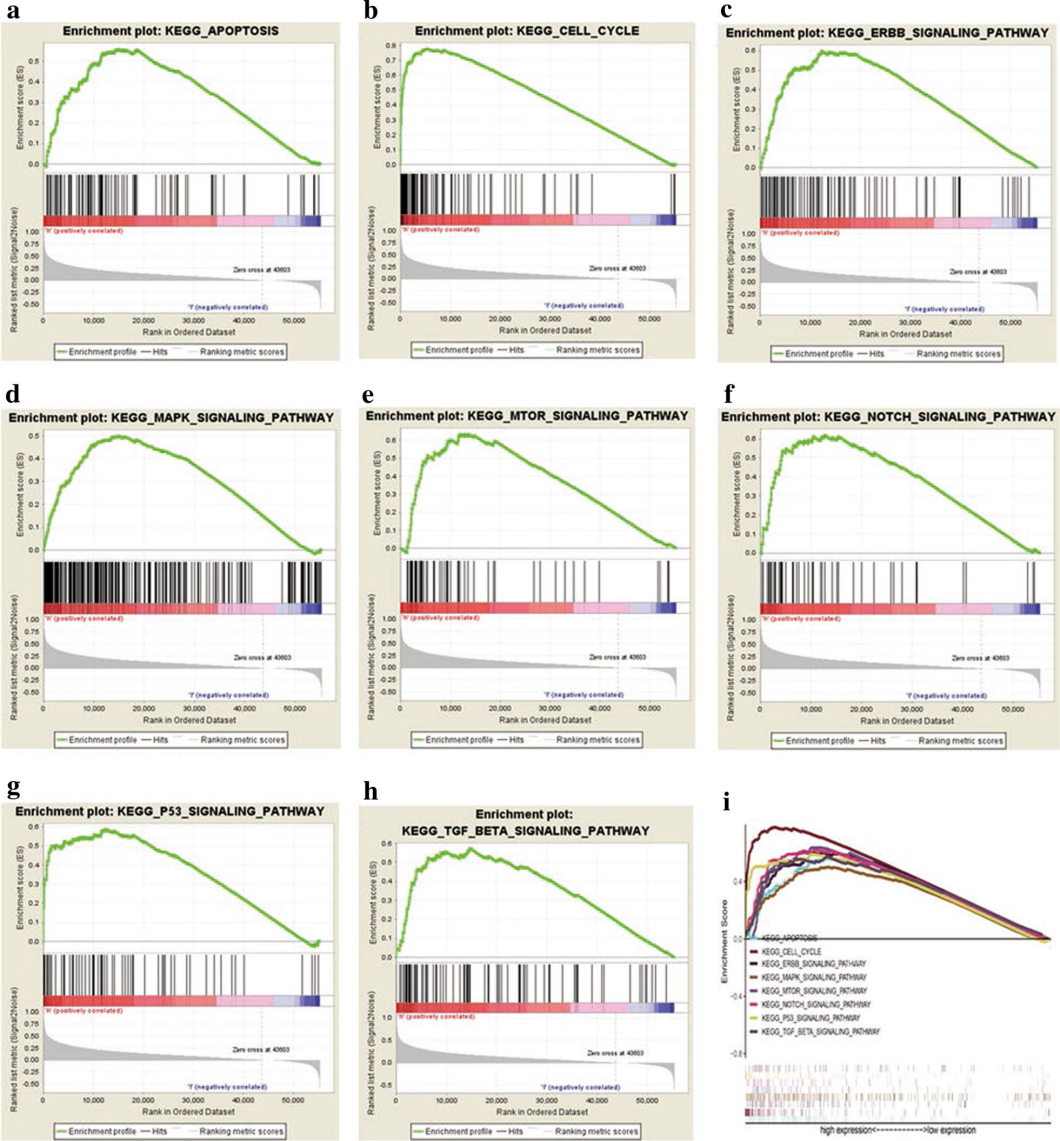
Enrichment plots from the gene set enrichment analysis (GSEA). GSEA results showing the apoptosis (**a**), cell cycle (**b**), ErbB signaling pathway (**c**), MAPK signaling pathway (**d**), mTOR signaling pathway (**e**), Notch signaling pathway (**f**), p53 signaling pathway (**g**),and TGF-beta signaling pathway (**h**). Multiple GSEA enrichment plots (**i**) of genes that are differentially enriched in *CDCA8*-related liver cancer. NES, normalized enrichment score; ES, enrichment score; FDR, false discovery rate

The GSEA results showed significant differences between the high and low *CDCA8* expression datasets based on the MSigDB enrichment analysis (c2.cp.kegg.v6.2.symbols.gmt). In the high *CDCA8* expression phenotype, the eight most significantly enriched signaling pathways (selected according to the NES) were the apoptosis, cell cycle, ErbB, MAPK, mTOR, Notch, p53 and TGF-β signaling pathways (Fig. 4, Table 4).

**Table 4:** Gene sets enriched in the high *CDCA8* expression phenotype

Gene set name	ES	NES	NOM *P*-val	FDR *Q*-val
KEGG_CELL_CYCLE	0.778	2.219	0.000	0.000
KEGG_P53_SIGNALING_PATHWAY	0.588	1.894	0.000	0.009
KEGG_MTOR_SIGNALING_PATHWAY	0.636	1.874	0.000	0.009
KEGG_ERBB_SIGNALING_PATHWAY	0.600	1.848	0.000	0.011
KEGG_TGF_BETA_SIGNALING_PATHWAY	0.575	1.829	0.000	0.013
KEGG_NOTCH_SIGNALING_PATHWAY	0.621	1.822	0.000	0.013
KEGG_APOPTOSIS	0.561	1.782	0.006	0.019
KEGG_MAPK_SIGNALING_PATHWAY	0.501	1.764	0.002	0.021

Gene sets with NOM *P*-value < 0.05 and FDR *Q-*valu*e* < 0.25 were considered significant ES, enrichment score; NES, normalized enrichment score; NOM, nominal; FDR, false discovery rate

## Discussion

*CDCA8* is a critical regulatory gene in mitosis. It plays an important role in different types of cancer, (e.g., promoting cell proliferation and invasion) and may act as an oncogene [12, 13]. Previous studies have reported the increased transcriptional activity of *CDCA8* in embryos, embryonic stem cells and cancer cells however, *CDCA8* is not expressed or is very weakly expressed in normal tissues [14]. Thus, the aberrant expression of *CDCA8* is strongly associated with cancer pathogenesis. Li et al. showed that *CDCA8* encodes the protein Borealin/Dasra B, which plays a critical role in regulating postnatal liver development, damage-induced hepatic progenitor-like cell regeneration, and liver tumorigenesis in mice [15]. These results suggest that *CDCA8* may impact the occurrence and progression of related liver diseases by modulating the function of the CPC in mitosis. However, only a few studies have explored the association between *CDCA8* and hepatitis, cirrhosis, and liver cancer. Our current study focused on the prognostic value of *CDCA8* in liver cancer.

Previous studies have reported that upregulated *CDCA8* expression plays an important role in malignant transformation, cancer growth and progression. Yu et al. showed that *CDCA8* induces tamoxifen resistance and promotes cell proliferation by inhibiting cell apoptosis and promoting cell cycle progression in breast cancer cells [16]. Ci et al. demonstrated that *CDCA8* knockdown inhibited cell proliferation, migration, and invasion in cutaneous melanoma cells via the Rho-associated coiled-coil-containing protein kinase (ROCK) signaling pathway [12]. Furthermore, *CDCA8* knockdown also inhibits cell proliferation and promotes cell differentiation in lung cancer, colorectal cancer, and human embryonic stem cells [8, 9, 17]. As described in the above studies, high *CDCA8* expression plays a key role in many types of cancer. Recently, an increasing number of studies have examined *CDCA8* as a potential prognostic marker. Gu et al. performed RNA-Seq data analysis and found that *CDCA8* is a prognostic gene in kidney renal clear cell carcinoma [18]. In addition, Ci et al. demonstrated that the overall survival of cutaneous melanoma patients with high *CDCA8* expression was significantly lower than that of patients with low expression, suggesting *CDCA8* as an independent predictor of prognosis in cutaneous melanoma [12]. Similar findings were previously observed in gastric cancer, lung cancer, breast cancer, and colorectal cancer [10, 19]. Consistent with these findings, our findings revealed that *CDCA8* expression was significantly upregulated in liver cancer tissues compared to matched normal tissues, indicating that the high expression of *CDCA8* is associated with the development of liver cancer. In addition, the increased levels of *CDCA8* expression in liver tissues were associated with an advanced T stage, an advanced clinical stage, a high histological grade, histological type and poor overall survival, suggesting that *CDCA8* is closely related to the malignant degree of liver cancer and predicts a poor prognosis for liver cancer. Cox model analysis demonstrated that high *CDCA8* expression was an independent prognostic factor in liver cancer, highlighting that *CDCA8* may be a potential biomarker for liver cancer prognosis.

In this study, we found that the *CDCA8* high expression phenotype was associated with the apoptosis, cell cycle, ErbB, MAPK, mTOR, Notch, p53 and TGF-β signaling pathways by GSEA. These pathways have been reported to be associated with the tumorigenesis, development and malignant phenotype of several cancers. Recently, many studies have shown that the occurrence and development of liver cancer involves the deregulation of several cellular signaling pathways. For instance, abnormal P53 expression is associated with concurrent acetylation and methylation at H3K27, which is associated with a more aggressive liver cancer cell tumour phenotype [20]. Liu et al. study showed that targeting the MAPK pathway has additive and synergistic effects when with other pathways important for liver cancer cell proliferation, such as the mammalian target of rapamycin (mTOR) and Wnt/β-catenin pathways [21]. The natural compound psilostachyin-A exerts its cytotoxic effects on liver cancer by blocking the ERK/MAPK pathway [22]. In addition, overexpression and aberrant signaling of the ErbB family of receptors have been implicated in liver cancer, but the mechanisms underlying ErbB overexpression are unclear [23]. Thus, these results indicate that *CDCA8* promotes cell growth and cancer metastasis and leads to poor survival in liver cancer patients through the above signaling pathways, and that *CDCA8* could serve as a new therapeutic target and prognostic marker in liver cancer. Further study is needed to elucidate the regulatory mechanisms.

Therefore, *CDCA8* overexpression is involved in the pathogenesis of several cancers and has potential value as a prognostic biomarker for liver cancer. However, our study still has some limitations. To fully elucidate the specific role of *CDCA8* in liver cancer, various clinical factors should be considered. Another limitation is the lack of such information or inconsistent data collection processes because the data were collected in different laboratories. Additionally, the data we analysed were derived from only a single public database. Hence, to avoid analysis bias caused by the retrospective nature of the current study, we should conduct prospective studies in the future. Finally, the current research is based on high-throughput gene sequencing data from the TCGA database. Therefore, we could not assay the expression of *CDCA8* with a single cell-based strategy, nor could we clearly assess the direct mechanism by which *CDCA8* is involved in the development of liver cancer. Therefore, further research, such as cell-based protein expression assays, is necessary to detect heterogeneity, and we will continue working hard to explore the direct mechanism of liver cancer.

## Conclusions

In conclusion, we found that the level of *CDCA8* expression was increased in liver cancer tissues and associated with a poor prognosis, suggesting that *CDCA8* may be a potential prognostic molecular predictor for liver cancer patients. Moreover, the apoptosis, cell cycle, ErbB, MAPK, mTOR, Notch, p53 and TGF-β signaling pathways may be related signaling pathways regulated by *CDCA8* in liver cancer.

## Supplementary Information

**Additional file 1: Table S1.** Characteristics of all patients with liver cancer from the TCGA database. Clinicopathological features of all liver cancer patients from the TCGA database.

**Additional file 2: Table S2.** Expression of *CDCA8* in normal tissues and liver cancer tissues.*CDCA8* expression is significantly higher in liver cancer tissues than in matched to normal tissues tissues in the TCGA dataset.

**Additional file 3: Table S3.** Kaplan–Meier survival analyses for OS analysis in liver cancer patients. Kaplan–Meier survival analysis was used to compare OS in liver cancer patients in the high and low *CDCA8* expression groups in liver cancer patients.

## Data Availability

Data and materials of this work are available from the corresponding author on reasonable request.

## Abbreviations

CDCA8: Cell division cycle associated 8
TCGA: The Cancer Genome Atlas
OS: Overall survival
CHOL: Cholangiocarcinoma
LIHC: Hepatocellular carcinoma
HR: Hazard ratio
CI: Confidence interval
GSEA: Gene set enrichment analysis
CPC: Chromosome passenger complex
MSigDB: Molecular Signatures Database
NES: Normalized enrichment score
ES: Enrichment score
FDR: False discovery rate
